# Three-Dimensional Surface Parameters and Multi-Fractal Spectrum of Corroded Steel

**DOI:** 10.1371/journal.pone.0131361

**Published:** 2015-06-29

**Authors:** Xu Shanhua, Ren Songbo, Wang Youde

**Affiliations:** School of Civil Engineering, Xi'an University of Architecture and Technology, Xi’an, Shannxi, People’s Republic of China; Medical University of Graz, AUSTRIA

## Abstract

To study multi-fractal behavior of corroded steel surface, a range of fractal surfaces of corroded surfaces of Q235 steel were constructed by using the Weierstrass-Mandelbrot method under a high total accuracy. The multi-fractal spectrum of fractal surface of corroded steel was calculated to study the multi-fractal characteristics of the W-M corroded surface. Based on the shape feature of the multi-fractal spectrum of corroded steel surface, the least squares method was applied to the quadratic fitting of the multi-fractal spectrum of corroded surface. The fitting function was quantitatively analyzed to simplify the calculation of multi-fractal characteristics of corroded surface. The results showed that the multi-fractal spectrum of corroded surface was fitted well with the method using quadratic curve fitting, and the evolution rules and trends were forecasted accurately. The findings can be applied to research on the mechanisms of corroded surface formation of steel and provide a new approach for the establishment of corrosion damage constitutive models of steel.

## Introduction

A rough surface with self-similarity and scale invariance always has a fractal geometrical property[1–2]. Many studies have been conducted on the morphology characteristics of rough surfaces on the basis of fractal geometry theory. Many types of fractal surfaces have been established and have played important roles in ultra-precision industries[3–6], materials science[7–10], electromagnetic wave scattering[11–13], and many other fields. In addition, in actual projects, the morphology of rough surface often presents variability, i.e., anisotropic and local characteristics in spatial distribution[14,15], which makes the fractal geometry theory difficulty to be applied in practical engineering.

As a common building material, steel, because of air-borne chloride ions, moisture, fugitive dust, etc., is highly susceptible to corrosion damage. The surface of corroded steel becomes gradually roughening from the very beginning of a plane. It well known, the surface of corroded steel is mainly roughened by corrosive pitting among all corrosion results, which is generating considerable interest. For geometric morphology, due to a large number of bumps or potholes (pits) and planar regions (without pits) on corroded surface of steel, the rough surface attacked by the corrosive pitting presents larger discreteness and concave convex feature. And the existing experimental data show that the multi-fractal dimension of a corroded steel surface is between two and three. The existing research results show that the surface attack by corrosive damage is characterized by continuity, non-differentiability and self-affinity, and is of multi-fractal features with a certain measure[16–18]. Thus, the research for the multi-fractal features is a powerful tool for analyzing the randomness and discreteness of the damage mechanism of corroded steel. However, the difficulties in the multi-fractal features analysis of corroded steel surface, such as tedious calculations, make it too complex to be applied to practical projects. Therefore, the present study is trying to use a simplified method to study the multi-fractal characteristics of corroded steel surface.

The Weierstrass-Mandelbrot (W-M) method is a common mathematical model used to construct the fractal surface in engineering. In this study, irregular fractal surface of the corroded steel is tried to be constructed by using the W-M method with specific fractal parameters, and calculate its multi-fractal spectrum. Then, based on the shape feature of the multi-fractal spectrum, the least squares method is applied to quadratic fitting of the multi-fractal spectrum of corroded surfaces. Finally, we conduct quantitatively analysis on the fitting curve to obtain an exact description and accurate analysis of the multi-fractal characteristics of the corroded steel surface.

## Materials and Methods

### Material and Specimens

**Fig 1:**
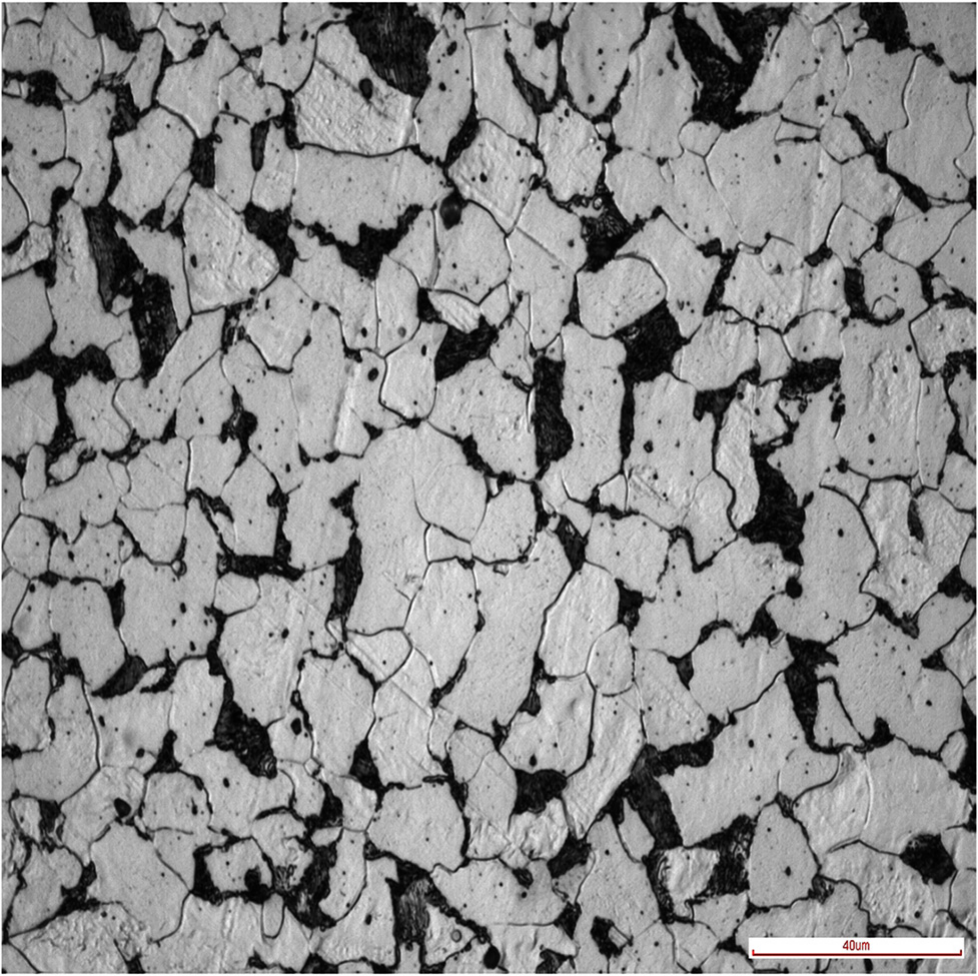
Microstructure of Q235 steel material. White part is ferrite and black part is pearlite.

The material used in this study was a Q235 steel, which is a normalized 0.25% low-carbon steel, extensively used in industrial and civil buildings: the matrix consists of a ferritic–pearlitic microstructure with a 20~40μm ferrite grain size, as shown in Fig 1; its basic chemical composition is shown in Table 1. All specimens were cut out from a same steel plate.

**Table 1:** Chemical composition of Q235 steel (wt. %).

C	Si	Mn	P	S	Cr	Ni	Cu	N
0.2	0.36	1.4	0.45	0.45	0.3	0.3	0.3	0.008

### Atmospheric Corrosion Experiment

**Fig 2:**
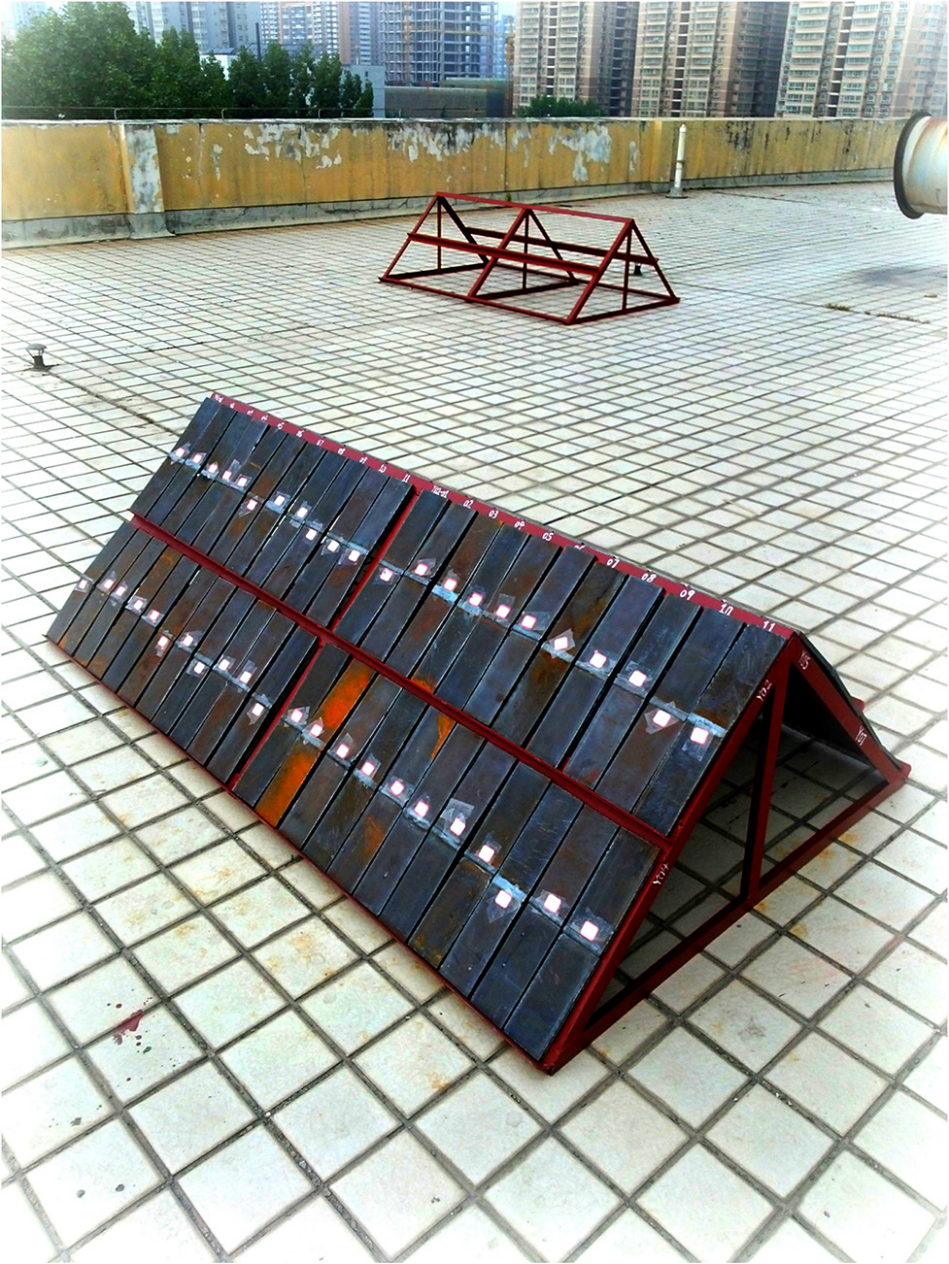
Setup of atmospheric exposure test.

A test sited in Xi’an (latitude 39°45' N, longitude 108°56' E) was selected for the atmospheric corrosion experiment. The test setup of the atmospheric corrosion experiment is shown in Fig 2. During the experimental period, the monthly temperature(*T*), relative humidity(*RH*), annual precipitation(*P*), and rainwater acidity(*PH*) values were measured to identify the atmospheric corrosive environment. According to GB/T 19292.4–2003[19], the specimens for corrosion were 400 mm in length, 60 mm in width and 8 mm in thickness, installed at 45°in the oblique direction. Table 2 presents the atmospheric corrosion conditions measured depending on the test site. After exposed for 0.5, 1, 2 and 4 years, orderly, the specimens were immersed in HCL solution (12% by volume) at room temperature for removing the corrosion products, cleaned with water, dried with hot air, rinsed in acetone, and then kept in a drying oven until the 3D profile measurement[20].

**Table 2:** Atmospheric corrosion conditions on the test site.

Exposed period	*T*(*K*)	*RH*(%)	*P*(mm)	*PH*
Mar. 2008-Apr. 2009	294	65	1755	6.3
Mar. 2009-Apr. 2010	294	61	2186	6.7
Mar. 2011-Apr. 2012	294	63	1738	6.4
Mar. 2012-Apr. 2013	295	69	2016	6.5
May	294	64	2106	6.6

*T* − the temperature; *RH* − the relative humidity; *P*− the annual precipitation; *PH*− the rainwater acidity.

### 3D Surface Profile Measurements

**Fig 3:**
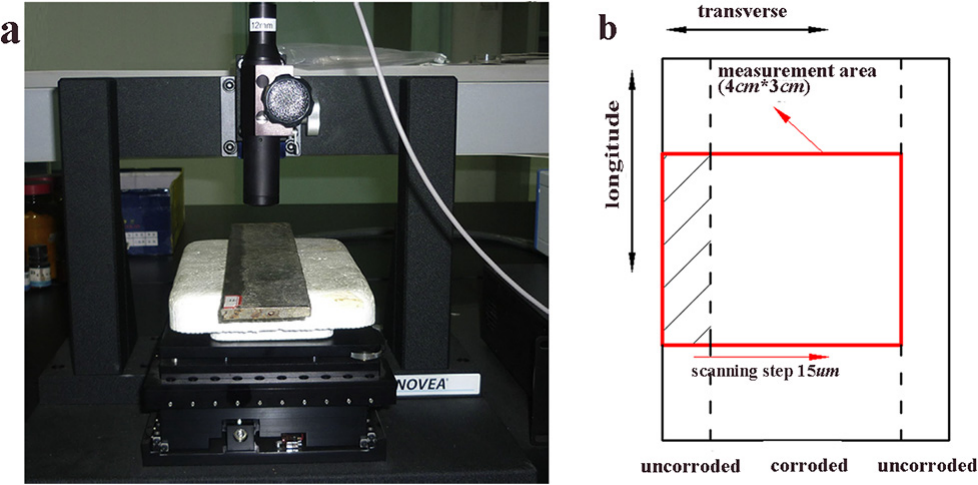
Laser focus measurement of the atmospheric exposure test specimen. (a) Equipment used for measurements; (b) Schematic diagram of the measurement area.

In order to gain the 3D morphology of the surface of corroded specimens, a non-contact PS50 3D profiler produced by NANOVER Company was used in this study. The 3D profiler was designed with leading edge white light axial chromatism optical pens to obtain nanometer resolution for surface inspection, hi-speed 3D metrology and more precise thickness mapping on a wider range of geometries and materials than any other profiometer[20].The vertical resolution was 0.28μm and the horizontal resolution was 8μm. The setup used for 3D surface morphology measurements is shown in Fig 3A. Each specimen had been detected the corroded surface at a measurement zone of 40 mm×30 mm(along the directions of transverse and longitude, respectively) approximately arranging at the central of specimen, and the scanning step was set to 15μm[20]. The elementary diagram of the measurement area is shown in Fig 3B.

### Surface Morphology and Pitting Characterization

**Fig 4:**
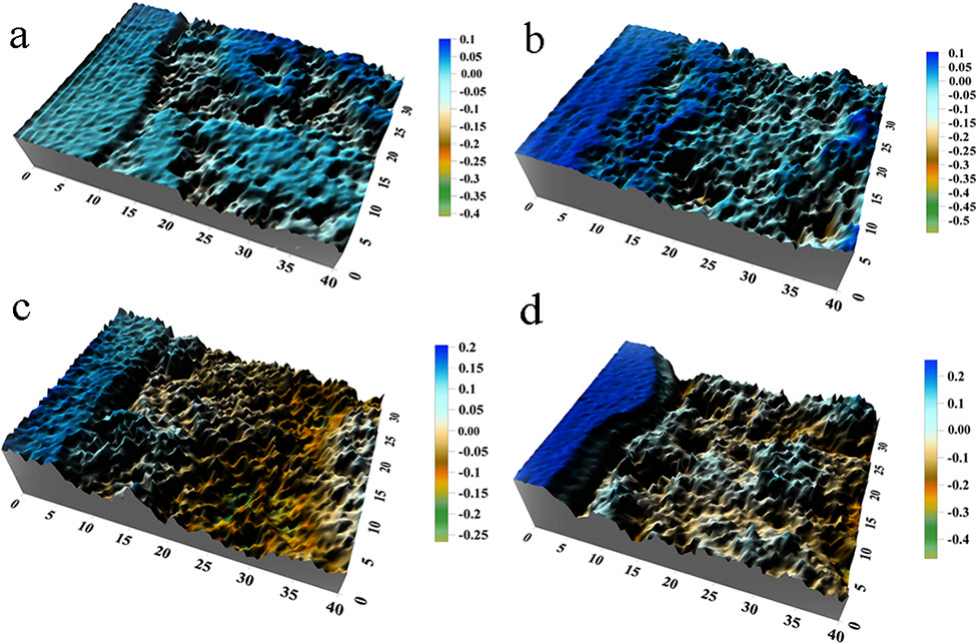
Contour of measured corroded surface images. (a) Surface image of 0.5 year old test specimen; (b) Surface image of 1 year old test specimen; (c) Surface image of 2 years old test specimen; (d) Surface image of 4 years old test specimen.

Fig 4 clearly shows the surface morphologies of corroded specimens exposed for 0.5, 1, 2 and 4 years, respectively. The images were draw by Golden Software Surfer 8. The software is a full-function 3D visualization, contouring and surface modeling package that runs under Microsoft windows, which is used extensively for terrain modeling, bathymetric modeling, landscape visualization, surface analysis, contour mapping, watershed and 3D surface mapping, gridding, volumetric, and much more. In this study, the 3D surface mapping was applied to show more detail of the corroded surface. The profiles of the different measurement regions are presented in Fig 4 (S1 Table).

It is well known, the corrosive attack can produce a network of corrosion on metal surface, which may be treated as a single pit or as two or more adjacent pits. Corrosion damage is also related to the link up of adjacent pits during subsequent exposure time. As shown in Fig 4, at the initial stage of corrosion, a few micro-pits only occurred on some particular locations on the corroded surface of specimens, which made the surface slightly irregular (shown in Fig 4A). And with the passage of corrosion time, large amounts of pits occurred on the surface and superimposed each other, which made the corroded surface signally irregular and uneven (shown in Fig 4D). Thus it can be seen that roughness and irregular of the corroded surface increased with the increasing exposure.

Although Fig 4 may directly reflect corrosion status of the corroded steel, the quantitative analysis from the angle of mathematics is hard to evaluate. Thus, the engineering application is in urgent need of a method, which can simulate the corroded surface of steel with high-precision and be convenient to quantitatively analyze the simulation.

## Results and Discussion

### Construction of the Multi-fractal Spectrum of Corroded Steel Surface

In science and engineering, many mathematical models with different multi-fractal characteristics based on the Weierstrass-Mandelbrot(W-M) method were generally used to analyze the surface conditions[21–26]. By using the W-M method, here, we can obtain a fractal surface with respect to an arbitrary surface of corroded steel through setting some specific multi-fractal parameters. For instance, when a fractal dimension (*D*) was known, the corresponding fractal surface can be obtained by employing the W-M fractal function, and the function is expressed as follows:

$$Z(x,y)=\sum_{n=1}^{\infty }C_{n}\lambda^{-{\left(3-D\right)}^{n}}\;\text{sin}\left[\lambda^{n}(x\;\text{cos}\;B_{n}+y\;\text{sin}\;B_{n})+A_{n}\right]$$ (1)

Where *C*_*n*_ is the characteristic length scale, i.e., the scaling constant; *n* is the wave number; *A*_*n*_ and *B*_*n*_, which have uniform distribution in [0, 2π], are independent random number, respectively; *D* is a multi-fractal dimension between 2 and 3; *λ* is constant greater than 1.

**Fig 5:**
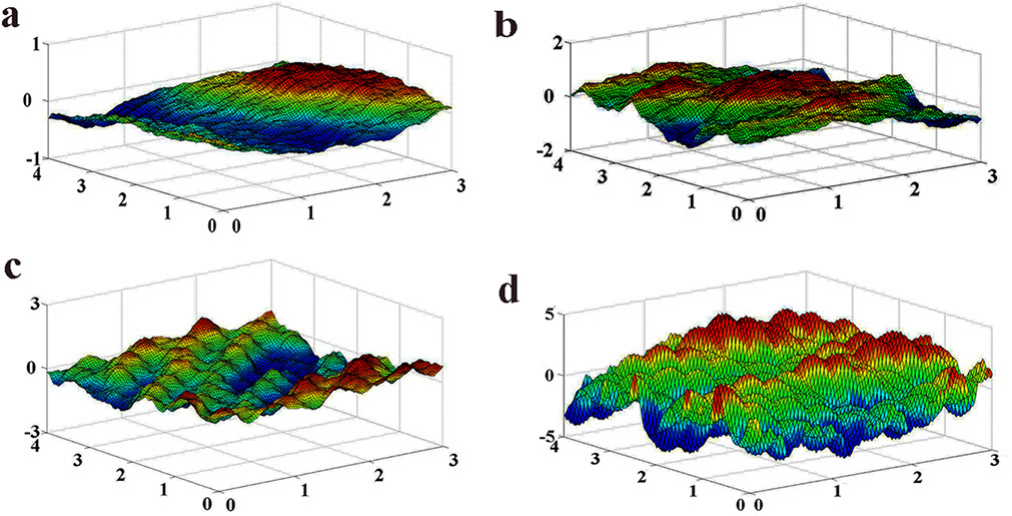
Fractal surfaces based on the W–M method. (a) Fractal surface with *D* = 2.2; (b) Fractal surface with *D* = 2.3; (c) Fractal surface with *D* = 2.5; (d) Fractal surface with *D* = 2.8.

In this study, the fractal surfaces with *D* value of 2.2, 2.3, 2.5, and 2.8 were constructed by the W–M method (*λ* = 1.3) to simulate the surface of corroded steel exposed for 0.5, 1, 2 and 4 years, respectively. Fig 5 shows the W–M surface with different fractal dimensions which were plotted by the MATLAB software.

**Fig 6:**
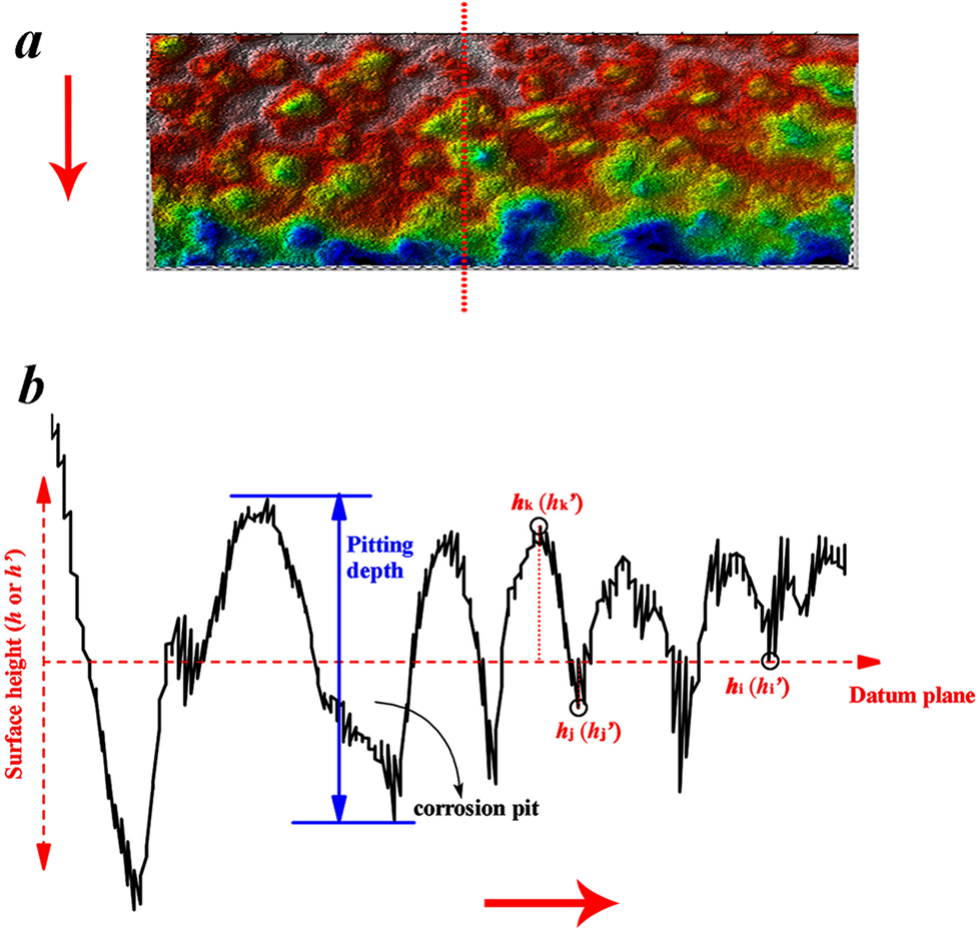
Surface profile (a) and the principle to obtain the surface height parameters (b), (red arrow indicates the trend of the profile). Blue indicates pitting depth, red indicates surface height based on the datum plane.

From Fig 5, it reveals that the fractal surface based on the W–M method was similar with the corresponding surface of corroded steel (shown in Fig 4) in roughness distribution pattern and surface fluctuation trend. Here we evaluated the accuracy of the constructed surface by comparing the measurement data and the theoretical data[27].

$$\mathrm{The simulated precision}=1-(\frac{\sum \left|h-h^{'}\right|}{h})\cdot \frac{1}{n}$$ (2)

where *h* is the measurement data of practical surface, *h* ^*’*^ is the theoretical data of simulated surface, *n* is the number of date. To obtain the surface height (*h* or *h* ^*’*^) of every point, the calculation principle was defined by using the method shown in Fig 6. Noted that the datum plane used to measure the height of the surface was obtained by least squares.

Table 3 showed the simulation precisions of fractal surfaces for corroded specimens with different corrosion time. And it can be found that the fractal surfaces with *D* value of 2.2, 2.3, 2.5, and 2.8 had more than 90% accuracy for simulating the corroded surfaces exposed for 0.5, 1, 2 and 4 years, respectively. That is to say, it is effective to simulate the rough surface of corroded steel by the W-M method.

**Table 3:** The simulation precisions of fractal surfaces for corroded specimens with different corrosion time.

Dimension	2.2	2.3	2.5	2.8
Simulation precision (%)	98.9	97.2	94.8	91.5

### Calculation of the Multi-fractal Spectrum

Many methods can be used to calculate fractal dimension[28–30], among which the multi-fractal dimension approaching to the real value can be obtained by the calculus of variations or the box counting method. Note that the box counting method, possessing the advantage of clear mathematical principle and simple calculation, was used in this study to calculate the multi-fractal parameters of corroded steel surface.

A set of boxes with size of *l* are used to divide the datum plane of the fractal surface of corroded steel**[** 31], make *ε* = *l* / *L*, *L* = 512 and *ε* < 1, *ν*_i_(*ε*) is distribution probability of the height of corroded surface for the box(*i*), which can be calculated as follow:

$$v_{i}(\varepsilon )=\frac{h_{i}}{\sum_{j}^{i}{\left(h_{i}\right)}_{j}}$$ (3)

In Eq (3), *h*_i_ is the height of corroded surface for the box(*i*), ∑(*h*_*i*_)_*j*_ is the sum of the height for all boxes. When the height distribution is of multi-fractal features, it can be described as:

$$v_{i}(\varepsilon )∼\varepsilon^{\alpha }$$ (4)

$$M_{\alpha }(\varepsilon )∼\varepsilon^{-f(\alpha )}$$ (5)

where *α* depending upon the box(*i*) is the singularity of the subset of height probabilities, M_*α*_(*ε*) refers to the number of boxes having the same height distribution probability when the size of box is *ε*, and *f*(*α*) is the multi-fractal dimension of subset with the *α* value. Generally, the value of M_*α*_(*ε*) increases with the decreasing of *ε*[32]. A *q*th-order partition function applied in statistical physics, *χ*_*q*_(*ε*), can be described as[33]:

$$\chi_{q}(\varepsilon )={\sum \nu_{i}(\varepsilon )}^{q}=\varepsilon^{\tau (q)}$$ (6)

$$\tau (q)=\underset{\tau \to 0}{\text{lim}}\left[\frac{\text{ln}\;x_{q}(\varepsilon )}{\text{ln}\;\varepsilon }\right]$$ (7)

where *q* is the moment order, *τ*(*q*) is a non-linear function of *q* and is known as the mass exponent function.

The singularity strength function *α* and the singularity spectrum *f*(*α*) can be calculated through Legendre transform:

$$\alpha (q)=\tau \prime (q)=\frac{d\tau (q)}{dq}$$ (8)

f(α(q))=q⋅α(q)−τ(q) (9)

Theoretically, with the increasing of |*q*|, the values of *α*(*q*) and *f*(*α*(*q*)) are closer to their theoretical limit. But in fact, when *q* reaches an oversized value, the computational workload will be increased significantly, which will lead to running out of memory; when |*q*| stands on an undersized value, of which the increment can cause obvious variation of *f*(*α*). The reason can be considered as that *f*(*α*) calculated through undersized |*q*| is only part of the multi-fractal spectrum, but cannot completely reflect probability distribution of the fractal surface. In practical, it is impossible to take the value of *q* to be infinite. But generally, the saturation extent of multi-fractal spectrum increases with the increasing of the *q*[31]; thus, we determined the value of *|q|*_*max*_ through that *f*(*α*) and *α* tend to be saturated. In this study, all of the multi-fractal spectrums of corroded surfaces were obtained with *|q|*_*max*_ = 60.

Through the analysis above, we calculated *f*(*α*)~*α* of the fractal surfaces (*D* = 2.2, 2.3, 2.5, and 2.8) corresponding to the corroded surfaces exposed for 0.5, 1, 2 and 4 years.

**Fig 7:**
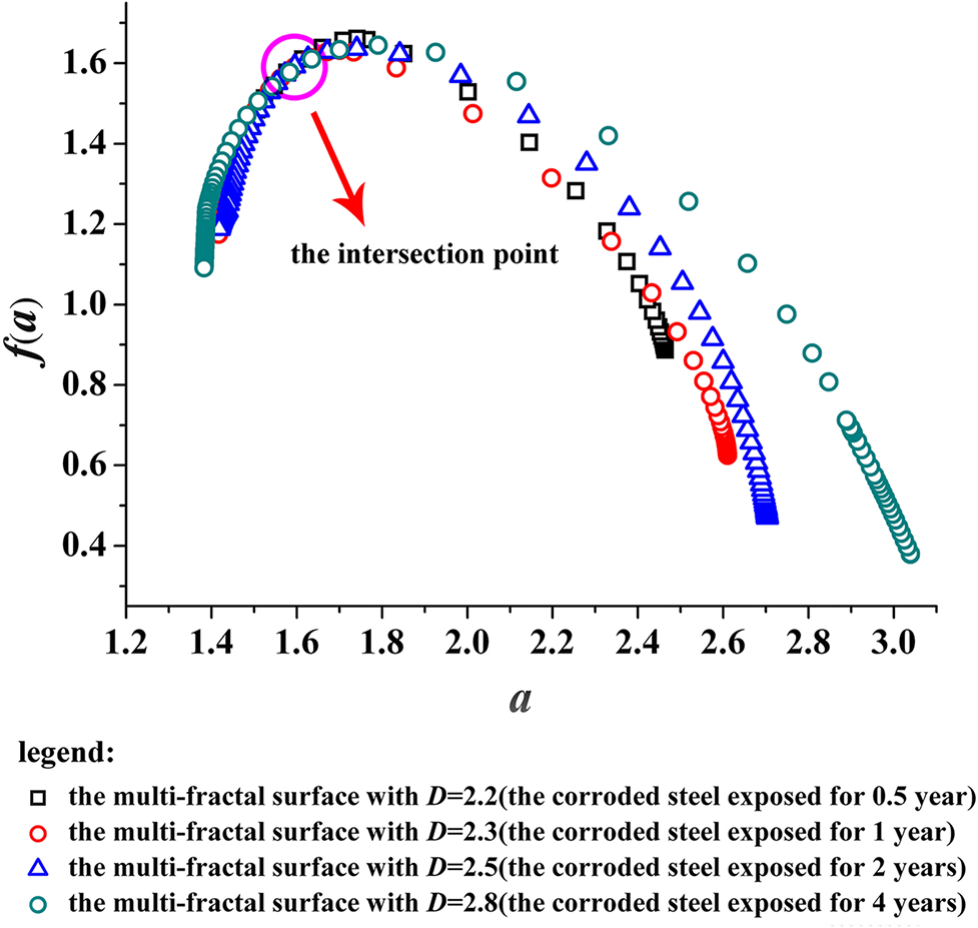
Multi-fractal spectrums, *f*(*α*)~*α*, with *D* = 2.2, 2.3, 2.5 and 2.8. The width of which along *α* denotes the degree of multi-fractal complexity.

Fig 7 plots the multi-fractal spectrums of the fractal surfaces with different dimensions (S2 Table). It can be seen that the shapes of all spectrums are inverted parabolas, but the difference of shapes among the spectrums is still obvious: the multi-fractal spectrum of corroded surface is plumping with the increasing of fractal dimension, and the spectrum span is also widening. It is well known value of Δ*α* (*α*_max_ − *α*_min_) determines the width of the spectrum, which is the main reason to increase the local indices of the studied variable, i.e., the more the unevenly distributing of surface, the wider the spectrum, the greater the heterogeneity of distribution and vice versa[34]. Thus, the conclusions can also be drawn: the surface of corroded steel unevenly distributes with the increasing of the value of Δ*α*.

Moreover, when the surface of corroded steel is more rough and irregular, the end position of the corresponding multi-fractal spectrum is lower, which means that the proportion of subsets referring to small probability continues to increase for the corroded surface.

As shown in Fig 7, the intersection point of all multi-fractal spectrums is (1.6, 1.6), which indicating the fractal subset with *α* = 1.6 (the edge of pits) has a largest proportion on the fractal surface. That is to say the fractal subset with *α* = 1.6 can determine the sharpness or roughness of the corroded steel surface, distinctly. For the Q235 steel under atmospheric corrosion environment, the corrosion damage mainly manifests corrosive pitting due to the heterogeneous microstructures and chemical composition[35, 36], as shown in Fig 4. Pits occur at some particular locations of the corroded surface, link up with adjacent pits with the increasing of exposure, and eventually influence the sharpness or roughness of the corroded surface. Thus, it can be seen the effect of atmospheric corrosion on surface condition of Q235 steel can be signally reflected by the development of corrosion pits with the method of multi-fractal.

### Fitting of the Multi-fractal Spectrum of Corroded Steel Surface

It can be known from Eq (8) and (9) that the calculation of multi-fractal spectrum is unable to be represented by using a specific or simple function, which makes the multi-fractal spectrum difficult to take quantitative analysis and to be applied in engineering. To simply formulate the multi-fractal spectrums of corroded surface, we had researched their trend and shape, and the following conclusion can be drawn:

Each multi-fractal spectrum (shown in Fig 7) can be decomposed into a left part and a right part of inverted parabolas and both of the parts are continuously differentiable.

Therefore, we can fit the multi-fractal spectrums of the fractal surfaces (with *D* = 2.2, 2.3, 2.5, and 2.8) corresponding to the corroded specimens exposed for 0.5, 1, 2 and 4 years by the method of least squares. And the left and right part of the multi-fractal spectrum can be respectively expressed as piecewise functions as follows for satisfying fitting precision in this study [37]:

$$f(\alpha (q))=A{\left[\left(\alpha (q)-\alpha_{0}(q\right)\right]}^{2}+B\left[\left(\alpha (q)-\alpha_{0}(q\right)\right]+C$$ (10)

where *α* is singularity strength by evaluating a probability of subset, *α*_0_ is the singularity strength for *D* = *D*_max_. *A*, *B*, and *C* are the undetermined coefficients, which can be obtained by a least squares procedure. Herein the absolute value of *A* is inversely proportional to the value of Δ*α*; the value of *C* is proportional to the maximum value of *f*(*α*).

Generally, the greater value of *α* can reflect a phenomenon that the probability of corresponding subset is smaller; the lower value of *α* can reflect a phenomenon that the probability of corresponding subset is bigger. The criterion reflected in multi-fractal spectrum can also be explained that the greater value of *α* can make the small probability of subset to be a significant impact on multi-fractal spectrum; on the contrary, the smaller value of *α* can make the big probability of subset to be a significant impact.

*f*(*α*) is a multi-fractal singularity spectrum representing irregularity and complexity of the fractal surface. The extreme values in the distribution of height probability are associated with the low values of *f*(*α*), *f*(*α*)_min_ and *f*(*α*)_max_, in such a way the big and small probability of height subsets are related to the left and right part of the spectrum, respectively.

Making comprehensive analysis of above discussions, the conclusions can be drawn: the greater the value of *C*, the higher the *f*(*α*)_max_, and the higher the *f*(*α*)_max_, the more complex and irregular the fractal surface of corroded steel; on the contrary, the smaller the value of *C*, the lower the *f*(*α*)_max_, and the lower the *f*(*α*)_max_, the more unobvious the complexity and regularity of corroded surface.

**Fig 8:**
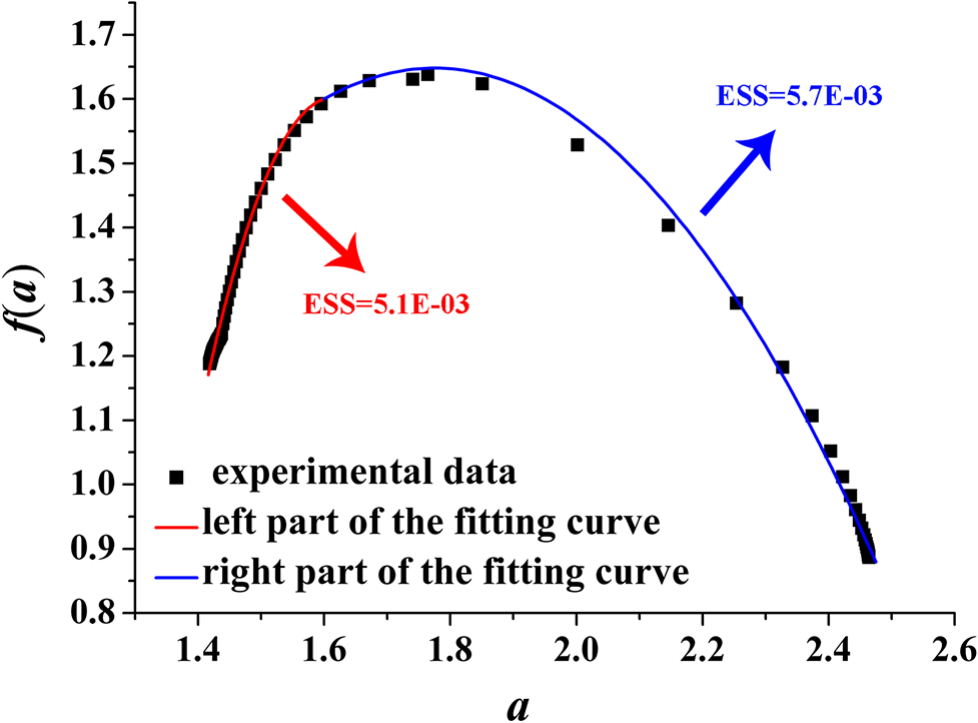
Left and right parts of the fitting curve of the multi-fractal spectrum with *D* = 2.2 (Eq 10). ESS is the error sum of square; red indicates the left part of the fitting curve; blue indicates the right part of the fitting curve. D-value of the minimum and maximum value is 1.05.

**Fig 9:**
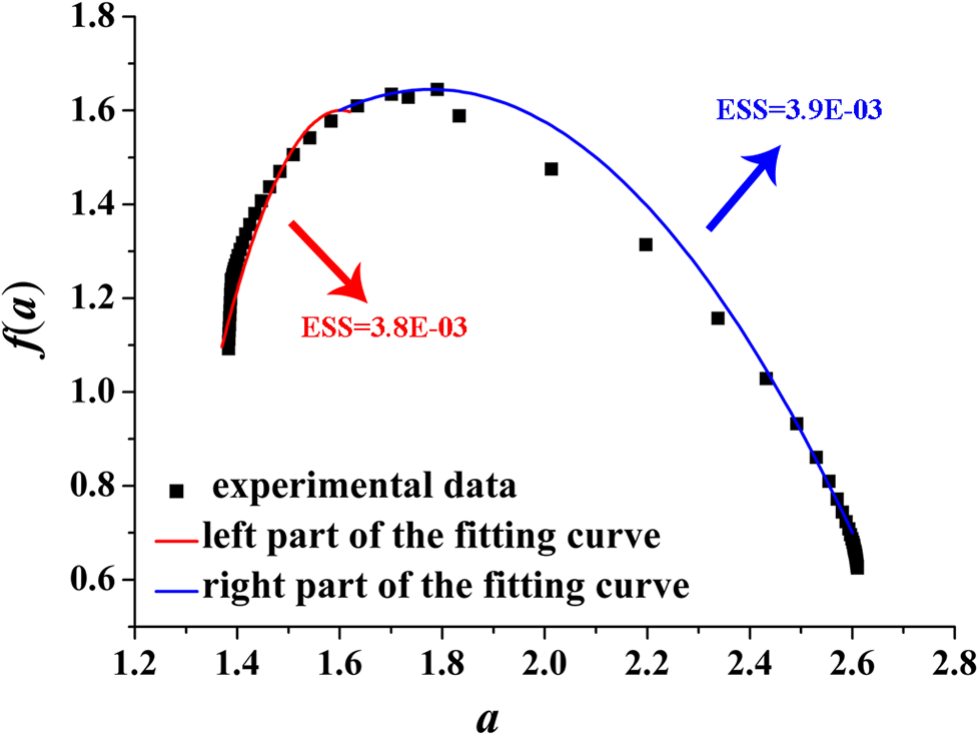
Left and right parts of the fitting curve of the multi-fractal spectrum with *D* = 2.3 (Eq 10). ESS is the error sum of square; red indicates the left part of the fitting curve; blue indicates the right part of the fitting curve. D-value of the minimum and maximum value is 1.23.

**Fig 10:**
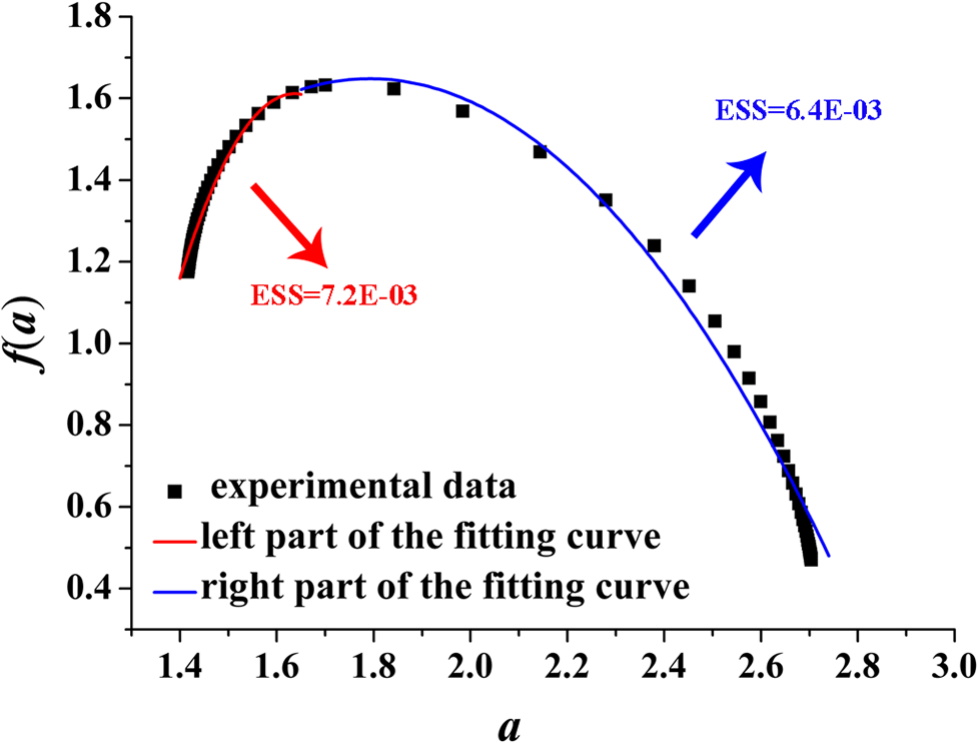
Left and right parts of the fitting curve of the multi-fractal spectrum with *D* = 2.5(Eq 10). ESS is the error sum of square; red indicates the left part of the fitting curve; blue indicates the right part of the fitting curve. D-value of the minimum and maximum value is 1.29.

**Fig 11:**
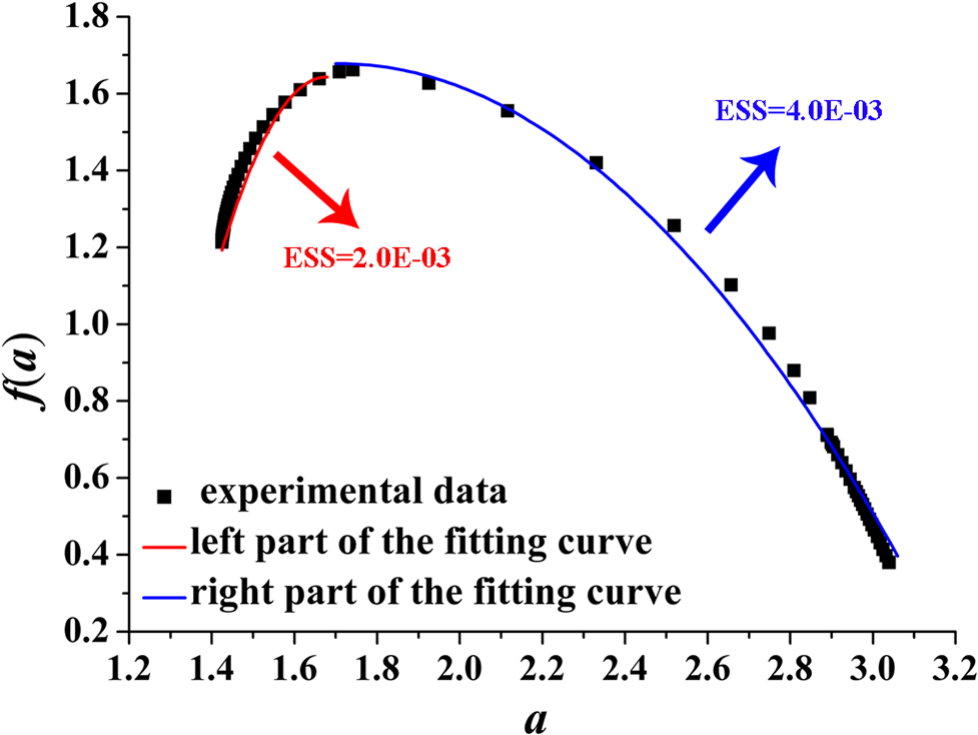
Left and right parts of the fitting curve of the multi-fractal spectrum with *D* = 2.8 (Eq 10). ESS is the error sum of square; red indicates the left part of the fitting curve; blue indicates the right part of the fitting curve. D-value of the minimum and maximum value is 1.61.

We fit *f*(*α*) of the right and left part by employing quadratic functions (red and blue curves shown in Figs 8–11) around *α*_0_ with least squares method, respectively.

Fig 8 plots the fitting curve of the multi-fractal spectrum with *D* = 2.2. The fitting functions of the right and left part curve are expressed as Eq (11) and Eq (12), respectively:

$$f(\alpha )=-1.57{\left(\alpha -1.6\right)}^{2}+0.55(\alpha -1.6)+1.6$$ (11)

$$f(\alpha )=-11.0{\left(\alpha -1.6\right)}^{2}+0.31(\alpha -1.6)+1.6$$ (12)

Fig 9 plots the fitting curve of the multi-fractal spectrum with *D* = 2.3. The fitting functions of the right and left part curve are expressed as Eq (13) and Eq (14), respectively:

$$f(\alpha )=-1.4{\left(\alpha -1.6\right)}^{2}+0.5(\alpha -1.6)+1.6$$ (13)

$$f(\alpha )=-9.3{\left(\alpha -1.6\right)}^{2}+0.55(\alpha -1.6)+1.6$$ (14)

Fig 10 plots the fitting curve of the multi-fractal spectrum with *D* = 2.5. The fitting functions of the right and left part curve are expressed as Eq (15) and Eq (16), respectively:

$$f(\alpha )=-1.3{\left(\alpha -1.6\right)}^{2}+0.5(\alpha -1.6)+1.6$$ (15)

$$f(\alpha )=-8.0{\left(\alpha -1.6\right)}^{2}+0.6(\alpha -1.6)+1.6$$ (16)

Fig 11 plots the fitting curve of the multi-fractal spectrum with *D* = 2.8. The fitting functions of the right and left part curve are expressed as Eq (17) and Eq (18), respectively:

$$f(\alpha )=-0.7{\left(\alpha -1.6\right)}^{2}+0.15(\alpha -1.6)+1.67$$ (17)

$$f(\alpha )=-7.0{\left(\alpha -1.6\right)}^{2}+1.1(\alpha -1.6)+1.6$$ (18)

Figs 8–11 show the fitting curves agreed well with computations which can meet the engineering precision requirements. According to the quantitative analysis of the fitting functions, we can find that the corroded surface of different specimen can be indeed reflected by a specific fitting curve of multi-fractal spectrum.

In multi-fractal spectrum, the left part determines the proportion of big probability subsets (the areas with obvious ups and downs) on the corroded surface; at this moment, the bigger the probability of subsets, the rougher the corroded surface, and the rougher the corroded surface, the smaller the value of the corresponding *α*. The right part determines the proportion of small probability subsets (the areas with smaller ups and downs); at this moment, the smaller the probability of subsets, the more smooth the corroded surface, and the smoother the corroded surface, the greater the value of the corresponding *α*.

Making intuitive observation of each fitting curve in Figs 8–11, |*A*| of left part curve is more than that of the right part, which explains the phenomenon that the range of the right part of the multi-fractal spectrum is larger than that of the left part. As well known, the larger the range of multi-fractal spectrum, the more the kinds of subsets described by the multi-fractal spectrum. Thus, it can be drawn that the subsets corresponding to the right part of the multi-fractal spectrum play a leading role in description of the multi-fractal spectrum.

Connecting with corrosion process of steel, when the steel surface is attacked by shallower pits in the initial stage of corrosion, |*A*| of the right part curve fitting the multi-fractal spectrum is larger and the corresponding range is narrower; the kinds of the subsets included in the corroded steel surface must also be less and the surface is almost a plane. With the increasing exposure time, because the pits contact the sub-surface constituent particles causing further corrosion and link up with adjacent pits damaging the corroded surface, |*A*| of the right part curve fitting the multi-fractal spectrum must be smaller and the corresponding range is wider; the kinds of the subsets including to the surface of corroded steel must also be more and the surface is more irregular and discrete.

Some parameter of the quadratic fitting is shown in Table 4. Making comprehensive analysis of Table 4 and Figs 4 and 5, the following conclusions can be made that with the increasing of Δ*α* from 1.05 to 1.61 or the decreasing of |*A*| from 11 to 7, the surface of corroded steel is distributing more unevenly and irregularly; with the increasing of Δ*f*(*α*) from 0.30 to 0.83, the surface will fluctuate more obviously.

**Table 4:** Parameters of the multi-fractal spectrum.

Dimension	*α*_min_	*α*_max_	Δ*α*	*α*_0_	*f*(*α*_min_)	*f*(*α*_max_)	*f*(*α*)_max_	Δ*f*(*α*)
2.2	1.42	2.46	1.05	1.60	1.19	0.89	1.60	0.30
2.3	1.38	2.61	1.23	1.60	1.09	0.62	1.60	0.47
2.5	1.42	2.70	1.29	1.60	1.18	0.47	1.60	0.70
2.8	1.42	3.04	1.61	1.60	1.21	0.38	1.60	0.83

*α*_min_− the minimum value of singularity strength; *α*_max_−the maximum value of singularity strength; Δ*α*–D-value of the minimum and maximum value; *α*_0_– the singularity strength for *D*_max_; *f*(*α*_min_)–the singularity spectrum for *α*_min_; *f*(*α*_max_)− the singularity spectrum for *α*_max_; *f*(*α*)_max_−the maximum value of singularity spectrum; Δ*f*(*α*)−D-value of the maximum and minimum spectrum.

It can be seen from the calculation formula of the multi-fractal spectrum, the weighting factor, *q*, is the slope of the multi-fractal spectrum, which can also be obtained using: *q* = ∂*f*(*q*(*α*)) / ∂*q*(*α*) [38]. For the multi-fractal spectrums mentioned above, whether the right part or the left part, an inequality exists as:

$$\frac{\partial q(\alpha )}{\partial \alpha }=\frac{1}{\left(\partial \alpha (q(\alpha ))/\partial q(\alpha )\right)}=\frac{1}{\left(\partial^{2}\tau (q(\alpha ))/\partial^{2}q(\alpha )\right)}<0$$ (19)

According to Eq (19), it can be seen *q* decreases with the increasing of *α*. In the left part of the fitting curve, *q* is a constant positive number; *q* is a constant negative number in that of the right part. |*q*| continues to increase with *α*. When *q* tends towards the plus infinity, the maximum probability subset has a significant impact on the multi-fractal spectrum, i.e., the corresponding corroded surface is a big bumps or potholes. Whereas *q* tends towards the minus infinity, the minimum probability subset has a significant impact on the multi-fractal spectrum, i.e., the corresponding corroded surface is similar to be a plane.

According to the analysis and discussion in this study, it can be known which part of subsets of the corroded surface has a decisive influence on shape and trending of the multi-fractal spectrum through mathematical analysis of fitting function of the multi-fractal spectrum. In addition, the overall shape and local characteristics of corroded surface of steel can also be derived by the analysis of fitting expression.

## Conclusions

In this study, we constructed the fractal surfaces of the corroded steel by using the W-M method and calculated the multi-fractal spectrums. Using the method of least squares to fit the multi-fractal spectrums of the fractal surfaces with *D* value of 2.2, 2.3, 2.5, and 2.8 corresponding to the surfaces of corroded specimens exposed for 0.5, 1, 2 and 4 years. Making comparison analysis of the fitting curves and calculated values, the following conclusions can be drawn:

1. *f*(*α*), between 1 and 2, was the fractal dimension of the subset relating to the singularity strength *α*, which was a measure of the complexity, irregularity and non-uniformity of the fractal surface. In physical condition, *f*(*α*) can intuitively reflect completeness of the fractal surface.
2. Δ*f*(*α*), *f*(*α*)_max_-*f*(*α*)_min_, reflected surface roughness; the higher the value of Δ*f*(*α*), the greater the difference among the subsets of the fractal surface of corroded steel, and the greater the difference among the subsets, the more irregular the fluctuation and distribution of corroded surface. On the contrary, for the fluctuation and distribution to be uniformity, the surface tends towards a plane.
3. *C*, given in the fitting function, can reflect the value of *f*(*α*)_max_ in the multi-fractal spectrum. The greater the value of *C*, the higher the value of *f*(*α*)_max_, and the higher the value of *f*(*α*)_max_, the more complex and irregular the fractal surface of corroded steel. Whereas, the smaller the value of *C*, the lower the value of *f*(*α*)_max_, and the lower the value of *f*(*α*)_max_, the better the completeness of the fractal surface.
4. *q* was constant positive number in the left part of the fitting curve and constant negative number in that of the right part. With the decreasing of *q*, the smaller probability subset had a significant impact on the multi-fractal spectrum; the description of the fractal surface of corroded steel is more subtle. This means that, with the decreasing of *q*, the subsets relating to the areas with slight ups and downs had a significant impact on the multi-fractal spectrum, which was conducive to represent more minutely the fractal surface of corroded steel and made the computed result much more close to the real value.

## Supporting Information

S1 Table The measurement data of corroded steel surfaces with different exposure time. *x*, *y* and *z* are geometric measurements in length, width and height, respectively. (XLSX).
(RAR)

S2 Table The multi-fractal spectrum of corroded steel surface. *α* is the singularity; *f* is the singularity spectrum; *D* is the fractal dimension. (XLSX).
(RAR)

## References

[1] Schroeder MR. Physics. Fractals, chaos, Power laws: Minutes from an infinite paradise 1st ed. Cambridge: Courier Dover Publication; 2012.

[2] Barabási AL. Fractal concepts in surface growth 1st ed London: Cambridge University Press; 1995.

[3] Tong XL, Li YH, Lin HS, Qi X. Research on phase-shifting interferometer contrast three-dimensional topography of ultra precision surface. Journal of Electronic Measurement and Instrument. 2009; 23(12): 65–69.

[4] Ren MJ, Cheung CF, Kong LB. A task specific uncertainty analysis method for least-squares-based form characterization of ultra-precision freeform surfaces. Measurement Science and Technology. 2012; 23(5): 54005–54014.

[5] Lee SR, Li ZG, Wang BG, Chiou HS. An application of the fractal theory in the design of heat sink for precision measurement instrument. Key Engineering Materials. 2005; 295: 717–722.

[6] Zong WJ, Sun T, Li D, Chen K, Dong S. Mechanical lapping single crystal diamond. Journal of Harbin Institute of Technology. 2005; 8(3): 285–288.

[7] Wan FX, Huang XP, Wu JF, Huang JL. Multi-fractal characteristic of metal material worn surface with plant abrasive. Applied Mechanics and Materials. 2014; 668: 43–47.

[8] Kamila AC, Juliana A, Thaís AP, Luis ROH. 3-D reconstruction by extended depth-of-field in failure analysis–Case study II: Fractal analysis of interlaminar fracture in carbon/epoxy composites. Engineering Failure Analysis. 2012; 25: 271–279.

[9] Lawrence KD, Ramamoorthy B. Structure function–based fractal characterisation of cylinder bore surfaces using stylus profile data. International Journal of Precision Technology. 2014; 4(1): 19–28.

[10] Ţălu Ş, Marković Z, Stach S, Marković BT, Ţălud M. Multifractal characterization of single wall carbon nanotube thin films surface upon exposure to optical parametric oscillator laser irradiation. Applied Surface Science. 2014; 289: 97–106.

[11] David B, Erik OS, Kimberly AR, Zachary DN, Andrew ST. Numerical prediction of sound scattering from surfaces with fractal geometry: A preliminary investigation. Ln: Engelhardt PV, Churukian AD, Rebello NS, editors. The Combined Use of Light and Sound for Imaging and Therapy. POMA 12: 161st Meeting Acoustical Society of America: 2011 May 23–27; Seattle, Washington. New York: American Institute of Physics; 2014. 12. p. 1–9.

[12] Eftaxia K, Frangos P, Kapiris P, Polygiannakis J. Kopanas J, Peratzakis A, et al Review and a model of pre-seismic electromagnetic emissions in terms of fractal electrodynamics. Fractals. 2004; 12(2): 243–273.

[13] Chen J, Lo TKY, Leung H, Litva J. The use of fractals for modeling EM waves scattering from rough sea surface. Geoscience and Remote Sensing; IEEE Transactions. 1996; 34(4): 966–972.

[14] Oleg VA, Dmitry NB, Alexander VK, Steen GH. Fractal description of rough surfaces. Applied optics. 2002; 41(22): 4620–4629. 12153095 10.1364/ao.41.004620

[15] Gan SY, Zhou Q, Xu XD, Hong YL, Liu Y, Fu SJ. Study on the surface roughness of substrate with multi-fractal spectrum. Microelectronic engineering. 2007; 84(5): 1806–1809.

[16] Berry MV, Lewis ZV. On the Weierstrass-Mandelbrot fractal function. Proceedings of the Royal Society of London A; 1980. pp: 459–484.

[17] El-Sonbaty IA, Khashaba UA, Selmy AI, Ali AI. Prediction of surface roughness profiles for milled surfaces using an artificial neural network and fractal geometry approach. Journal of Materials Processing Technology. 2008; 200(1): 271–278.

[18] Majumdar A, Tien CL. Fractal characterization and simulation of rough surface. Wear. 1990; 136(2): 313–327.

[19] Corrosion of metals and alloys-corrosively of atmospheres-Determination of corrosion rate of standard specimens for the evaluation of corrosively. GB/T 19292.4–2003.

[20] Xu SH, Wang YD. Estimating the effects of corrosion pits on the fatigue life of steel plate based on the 3D profile. International Journal of Fatigue. 2015; 72: 27–41.

[21] Qi DW. Analysis and processing of X-ray image of log with defects based on fractal theory. Ph. D. Thesis, Northeast Forestry University. 2003. Available: http://cdmd.cnki.com.cn/Article/CDMD-10225-2003110997.htm.

[22] Jiang Z, Wang H, Fei B. Research into the application of fractal geometry in characterizing machined surfaces. International Journal of Machine Tools and Manufacture. 2001; 41(13): 2179–2185.

[23] Liao XY, Lei WY. The geometric precision and performance analysis for matching surface based on fractals. Journal of Chongqing University. 1999; 22(6): 18–23.

[24] Mandelbrot BB. Stochastic models for the earth's relief, the shape and the fractal dimension of the coastlines, and the number-area rule for islands. Proceedings of the National Academy of Sciences USA. 1975; 72(10): 3825–3828. 10.1073/pnas.72.10.3825 PMC433088 16578734

[25] Mandelbrot BB. The Fractal Geometry of Nature 1st ed. New York: Macmillan Publishers; 1983.

[26] Bora CK, Flater EE, Street MD, Redmond JM, Starr MJ, Carpick RW. Multi-scale roughness and modeling of MEMS interfaces. Tribology Letters. 2005; 19(1): 37–48.

[27] Sun HQ, Xie HP. Fractal simulation of rock fracture surface. Rock and Soil Mechanics. 2008;28(2): 347–352.

[28] Ni HJ, Huang XL, Ning XB, Huo CY, Liu TB, Ben D. Multi-fractal analysis of resting state fmri series in default mode network: age and gender effects. Chinese Science Bulletin. 2014; 59(25): 3107–3113.

[29] Yang XD, He AJ, Zhou Y, Ning XB. Multi-fractal mass exponent spectrum of complex physiological time series. Chinese Science Bulletin. 2010; 55: 1996–2003.

[30] Vahedi A, Gorczyca B. Settling velocities of multi-fractal flocs formed in chemical coagulation process. Water Research. 2014; 53: 322–328. doi: 10.1016/j.watres.2014.01.008 24530551 10.1016/j.watres.2014.01.008

[31] Sun X, Wu ZQ, Huang Y. Fractal theory and its applications 1st ed. Hefei: China Science and Technology University Press; 2003.

[32] Sayyad AJ, Nikooee E, Ayatollahi S, Alamdari A. Investigating wettability alteration due to asphaltene precipitation: Imprints in surface multi-fractal characteristics. Applied Surface Science. 2013; 256(21): 6466–6472.

[33] Bird N, Díaz MC, Saa A, Tarquis AM. Tarquis. Fractal and multi-fractal analysis of pore-scale images of soil. Journal of hydrology. 2006; 322: 211–219.

[34] Zeleke TB, Si BC. Characterizing scale-dependent spatial relationships betweensoil properties using multi-fractal techniques.Geoderma. 2006; 134: 440–452.

[35] Du CW, Li XG, Liang P, Liu ZY, Jia GF, Cheng YF. Effects of microstructure on corrosion of X70 pipe steel in an alkaline soil. Journal of materials engineering and performance. 2009; 18(2): 216–220.

[36] Wang LW, Du CW, Liu ZY, Zeng XX, Li XG. Influences of Fe_3_C and pearlite on the electrochemical corrosion behaviors of low carbon ferrite steel. ActeMetallurgicaSinica. 2011; 47(10): 1227–1232.

[37] Smirnov VI. A course of higher mathematics 1st ed. London:Pergamon Press; 1964.

[38] Cai XQ. The statistics of multi-fractal parameter. Journal of Zhangzhou Teachers College.1997; 3: 109–113.

